# An Integrative Review of the Barriers to Indigenous Peoples Participation in Biobanking and Genomic Research

**DOI:** 10.1200/JGO.18.00156

**Published:** 2019-03-07

**Authors:** Jaclyn Aramoana, Jonathan Koea

**Affiliations:** ^1^ North Shore Hospital, Takapuna, Auckland, New Zealand

## Abstract

**PURPOSE:**

This investigation was undertaken to define the barriers to Indigenous peoples participating in biobanking and genomic research.

**METHODS:**

A literature review was conducted to identify studies reporting on the experience of Indigenous peoples with biobanking, tissue banking, and genomic research. Studies pertaining to organ transplantation or blood donation for transfusion were excluded. The databases searched were MEDLINE, EMBASE, PubMed, Web of Science, and Google Scholar, with all literature available until the search date of June 1, 2018, included. The reference lists of all included papers, as well as related review articles, were manually searched to identify additional relevant studies. An inductive approach was used to identify common themes.

**RESULTS:**

Seventeen publications discussed the experiences of New Zealand Māori (n = 2), Aboriginal and Torres Strait Islanders (n = 3), Native Hawaiian (n = 4), Native Alaskan (n = 2), American First Nation (n = 2), or multiple ethnicities (n = 4). Across all Indigenous peoples, four themes emerged: land, ancestors, culture, and bodily substances are powerfully interconnected and can act on each other; tissue and blood can provide important information (both Western and traditional) about a person; the ownership of specimens—custodians, trustees, or guardians; and the beneficence of the researchers and research team.

**CONCLUSION:**

Indigenous communities, like Western populations, are concerned with issues pertaining to handling, treatment, and ownership of tissue as well as knowledge gained from specimen analysis. Unlike many Western populations, Indigenous communities have retained a strong sense of cultural connection to ancestors and traditional lands and view biologic specimens as inseparable from these things.

## INTRODUCTION

Research involving human biospecimens and genetic information, in conjunction with clinical and treatment outcome data, has become increasingly important in determining the causes and potential treatment strategies for many complex diseases. Consequently, many universities and medical institutions have developed and administer biobanks, whose purpose is to collect and store human genetic materials (ranging from blood to abnormal and normal tissue biopsy specimens) and their respective clinical data.^1,2^ Specimens may be collected for a single research protocol or, more commonly, collected and consented for storage for future investigations.

Indigenous peoples are defined as the ethnic groups who are the original inhabitants of a given region, in contrast to groups who have settled, occupied, or colonized the area more recently.^3^ Populations are usually described as Indigenous when they maintain traditions or other aspects of an early culture associated with a given region. Many Indigenous populations do not suffer from the same diseases in the same ratios as observed in European populations and, although social determinants exert a significant influence on the health of Indigenous communities, study of the genome of Indigenous peoples, especially in geographic isolates, may clarify significant risk and protective factors for some conditions.^4^ However, attitudes to and participation in biobank research differ by culture and ethnicity, with Indigenous, nonwhite populations less likely to participate in biobank research and more likely to express concerns about collection, storage, and use of biospecimens.^5^ The reasons for this reluctance to participate in biobanking research are numerous and relate to cultural differences between research teams and target populations and past negative experiences of research conduct and state-run organizations.^6^

A number of investigators have previously published guidelines for researchers looking to successfully interact with specific Indigenous communities^3^; however, no formal global synthesis of Indigenous peoples’ views on this topic has been established. This integrative review was undertaken with the aim of comprehensively reviewing Indigenous peoples’ views on the barriers they face to participation in biobanking and genomic research and advancing potential solutions for both Indigenous communities and researchers.

## METHODS

### Literature Search

**FIG 1:**
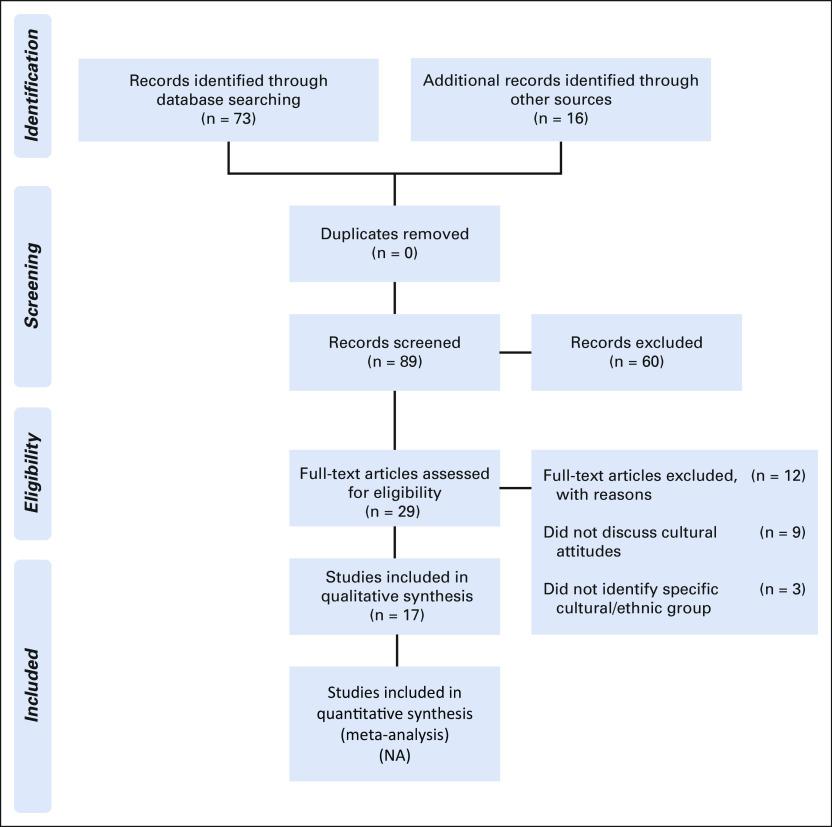
PRISMA^6^ flow diagram. NA, not applicable.

The search terms used were: “tissue,” “tissues,” “biobank,” “bio bank,” “tissue bank,” “tissue banking,” “culture,” “biologic material,” “cultural,” “perceptions,” “Indigenous,” and “attitudes.” The databases examined were MEDLINE, EMBASE, PubMed, Web of Science, and Google Scholar, with all literature available on these data sets through the search date of June 1, 2018, included. There were no language or geographic limits. The reference lists of all included papers, as well as related review articles, were manually searched to identify additional relevant studies. After the literature search, titles were screened for relevance, followed by review of the abstract (n = 89 manuscripts) and a full-text read to determine if studies were to be included (n = 60). A flowchart indicating the number of manuscripts excluded at each step is shown in Figure 1.^6^

### Study Selection

Studies that reported on the experience of Indigenous peoples with biobanking, tissue banking, blood collection, and genomic research were included. Studies were excluded if they reported only scientific results of investigations carried out in Indigenous communities and did not present the viewpoint of Indigenous communities or Indigenous participants. Studies focusing on organ transplantation and blood donation for transfusion were also excluded. Publications were reviewed for inclusion independently by the two authors (J.A., J.K.), with disagreement resolved by consultation.

### Risk of Bias Assessment

Critical appraisal of the selected studies was performed using the Effective Practice and Organization of Care (EPOC) risk of bias tool.^7^ This tool contains nine standardized criteria and scores the risk of bias as low, unclear, or high, both within and between studies.

### Data Abstraction and Analysis

A general inductive approach was used to systematically examine the literature. Each reviewer (J.A., J.K.) read the text thoroughly several times. Basic details of included studies, such as sample size, study design, participant type, country of origin, and stated aim, were recorded, tabulated, and rated according to the Oxford Centre for Evidence–Based Medicine.^8^ The process of reviewing the texts multiple times allowed identification of themes that were reflected across some or all the included studies. The themes were summarized, and if multiple studies reported the same phenomenon, a saturation effect was considered likely. An attempt was made to identify a relationship between the different themes to see how they were connected, which was explored more in the discussion. The data were interpreted along with data from a literature review to seek a justification for the findings.

## RESULTS

### Study Characteristics

The review of initial potential papers from abstracts, titles, and reference lists of retrieved papers resulted in a total of 89 papers. Sixty were excluded on review of the abstract, against the inclusion criteria. The remaining 29 papers were then assessed, with an additional 12 being excluded for the following reasons: nine did not discuss cultural attitudes to tissue banking and three did not identify the specific culture or group of people being studied (Fig 1), leaving 17 publications included in this review.^3,9-24^ The included publications described the experiences of Australian Aboriginal and Torres Strait Islanders,^10,16,19^ New Zealand Māori,^3,9^ Native Hawaiian,^13,20,21,23^ Native Alaskan,^14,15^ and North American First Nation,^12,22^ and multiple ethnic groups.^11,17,18,24^

### Methodological Quality

The 17 papers were composed of studies that used focus group methods to collect data,^13-15^ conducted semistructured interviews with individuals,^9,12,21^ and used mixed methods (individual and focus groups),^10,11,16,19^ surveys,^20^ and community viewpoints or summary documents.^3,17,18,22-24^ Using these investigations, four themes were identified, with Table 1 summarizing the studies and Table 2 providing select quotations and results illustrating the themes.

**TABLE 1:**
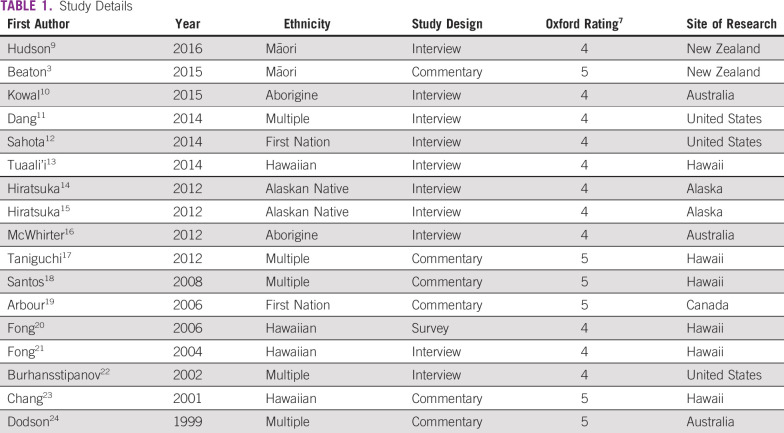
Study Details

**TABLE 2:**
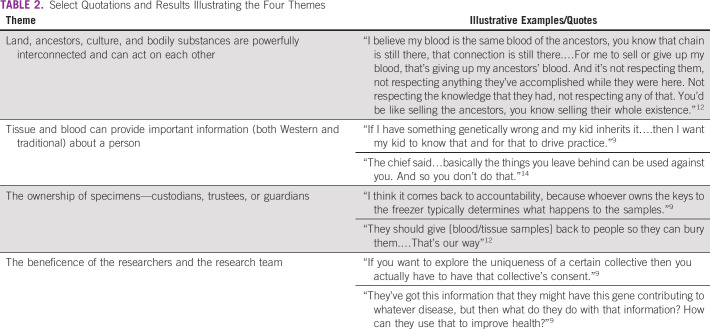
Select Quotations and Results Illustrating the Four Themes

### Risk of Bias Characteristics

On the basis of the EPOC tool^7^ results, 11 investigations had a low risk of bias within the study,^9-16,20-22^ and six had an unclear risk of within-study bias.^3,17-19,23,24^ All 17 investigations were assessed as having a low risk of a cross-study bias.^3,9-24^

### Theme 1: Land, Ancestors, Culture, and Bodily Substances Are Powerfully Interconnected and Can Act on Each Other

Kowal et al^10^ emphasize that, in Australian Aboriginal culture, land, ancestors, living individuals, and all bodily substances are interconnected and can act positively or negatively on each other. Almost identical beliefs are seen in New Zealand Māori,^9^ Hawaiian,^13^ and First Nations peoples,^12^ as well as in many world religions. Similarly, Aboriginal culture is infinite, dating from the deep past and proceeding into the future,^10^ and this sense of all individuals being part of a much greater and ancient continuum is also seen in other Indigenous peoples.^9,12,13^ For Indigenous people and researchers, these beliefs have important implications. First, if culture is infinite, preservation of tribal property^24^ and the maintenance of traditions may be more important than present events and demands,^24^ and consequently the extended family and tribal grouping are of greater importance than any individual or any nuclear family.^24^ Second, bodily health is not separate from the health of the culture, the land, and the community, so issues with handling of tissue and blood can adversely affect community culture and well-being. This is particularly important when it is appreciated that many Indigenous peoples are currently living in poverty associated with loss of traditional lands and grappling with the cultural and social consequences of that loss.^24^ First Nations peoples’ belief is that body parts retain the essence of the individual even after removal from the body and retain elements of ancestors, and, therefore, to sell or give away blood or tissue is to give up a link to ancestors.^12^ Wholeness of the body extends to signify completeness in a social and cultural context. The connection of body to ancestral lands is also important in specimen storage and disposal. Removal of specimens from the country and, at times, the region of origin is often not permitted by Indigenous belief, and any attempt to remove tissue (or derived DNA or RNA) for overseas analysis must be specifically dealt with in the consent process.^3,9^

These Indigenous cosmologic beliefs are often different from those current in contemporary Western society and well outside the perspective of many Western academic researchers. In 2001, in response to the Human Genome Project, Chang et al^23^ reviewed laws in Hawaii and reached the conclusion that, at that time, there was inadequate protection for Native Hawaiians in the matter of genetic research. Burhansstipanov et al^22^ then proposed a participatory research design with researchers and communities working closely to clarify which questions, of high health priority to the Indigenous community and to the researchers, should be addressed and how this should be undertaken. Subsequently Taniguchi et al^17^ have summarized contemporary research practices and research law in Australia, New Zealand, Canada, and the United States. In this comparative analysis, these investigators found that both Australia and New Zealand did not mandate for early community consultation, provisions for community withdrawal, and reconsenting for secondary investigations. In addition, there was no clear position on benefits accruing to the community from research findings or their commercial application. This has meant that ethics committees and institutional review boards in all four countries often lack clear metrics to assess the impact of biobanking and genomic investigations on potentially vulnerable Indigenous communities,^3^ and Taniguchi et al^17^ have advocated for an internationally developed and accepted legislative framework to protect all Indigenous communities.

### Theme 2: Tissue and Blood Can Provide Important Information (both Western and traditional) About a Person

Blood and tissue are highly symbolic in the cultures of many Indigenous peoples and often play important roles in traditional ceremonies.^10^ The acknowledged link of tissue and blood taken from present-day community members with ancestors means that all Indigenous communities appreciate that blood and tissue are an important source of information about individuals and their communities.^10^ In addition, although viewed as renewable from a Western perspective, blood is finite and cannot be created—only exchanged—between body, land, and spiritual entities.^22^ Consequently, blood and tissue must be treated as precious and handled in a culturally appropriate way. This may require researchers to create expert advisory panels^22^ from local experts to ensure that appropriate specimen management takes place. Indigenous communities have previously noted that culturally inappropriate handling of tissue and blood samples has often meant that donor families have to correct transgressions with a time-consuming and expensive ceremony.^12^

Information obtained from Indigenous blood and tissue must also be interpreted with care and used for the purpose for which individual and community consent was provided. Blood from the Havasupai people of North America was taken for diabetes research but was used for investigations into schizophrenia and migration studies without tribal consent.^18^ Previous investigations have used blood to determine levels of genetic purity in First Nations peoples and to trace migration patterns. For the First Nations peoples, this has often threatened tribal sovereignty and rights.^22^ For Alaskan Natives, who are recognized by blood quantum,^14,24^ this has threatened both individual and tribal rights to resources as specified under the Dawes Act.^12^ In addition, using genetic data to define potential phenotypes is fraught when the relationship between environment and genotype is not fully understood. Two commonly quoted examples of this phenomenon have been in the association between Aboriginal genetic markers and alcoholism^24^ and the presence of a warrior gene for aggression in New Zealand Māori.^3,9^

Finally, the link between an ancestral past and present and the significance of blood and tissue for Indigenous peoples makes genetic manipulation and the immortalization of cell lines deeply concerning to Indigenous communities.^24^ If planned, this must be explicitly discussed during study development. Similarly, one-fifth of human genes have been patented in the United States,^19^ and Indigenous people are opposed to patenting part of a life form that cannot claim to be invented.^18,19^

### Theme 3: The Ownership of Specimens—Custodians, Trustees, or Guardians?

None of the Indigenous peoples included in this review developed societies where land and other property resided in individual ownership and could be sold.^9,10,12,24^ This has meant that the optimal ownership model for Indigenous biologic specimens has often been confused. Because of the significance of blood and tissue to Indigenous communities and previous experiences, such as those of the Havasupai, where specimens have not been used for their stated purpose,^18^ Arbour and Cook^19^ have advanced the concept of DNA on loan, where tissue is loaned to the research team for a specific purpose. Once completed, the community, individuals, and the researchers retain the ability to renegotiate future uses of the specimens. Arbour and Cook^19^ emphasize that data from the research should also be considered as mutually owned by the community and research group or, at least, agreed to as owned by the community but under the stewardship of the researcher.^19^ Most importantly, whatever model is used, the Indigenous community must have ongoing input and the ability to influence specimen use and disposition. This should also be independent of the type of specimen; samples like placenta, fingernails, hair, and urine may be viewed as waste in European society but have significance and importance in Indigenous communities.^21^ Fong et al^21^ have shown that 78% of Hawaiians want to be reconsented for reuse of identifiable specimens, and 35% want to be reconsented for anonymized specimens. This finding challenges the common rule, which does not require researchers with anonymized specimens to recontact donors for new research use, on the premise that if donors cannot be identified then they cannot be harmed. However, this rule is dependent on a Western rather than an Indigenous concept of harm.^12,21,23^ Guidelines from Alaska request clear consent on what specimens will be used for and a destroy date or destroy option that can be activated in the event of a donor’s death,^15^ as does the Paoakalani Declaration from Hawaii.^18^

The use of anonymized specimens in Indigenous research is also noteworthy. Many Indigenous communities want the results of research reported to individuals and the community^21^ regardless of whether the samples are clinically or research derived. In addition, Indigenous communities are more likely to engage in biobanking and genomic research if someone they know or the community is more likely to benefit directly,^19^ and deidentified specimens may mean that this does not occur.^14^

### Theme 4: The Beneficence of the Researchers and Research Team

The term beneficence was popularized by Hiratsuka et al^15^ as a measure for how much emphasis the researcher and research team place on the welfare of the Indigenous community in the research process. This was in response to early research projects that were deemed extractive,^18^ in that data were simply removed from communities for the benefit of the researchers without any reference to, or improvement in, community life. In this model, researchers developed projects without consideration of the needs of communities^22^ and took no responsibility for improving the health and status of Indigenous communities, simply maintaining the status quo, which was usually a reality of poverty and deprivation.^18,24^ Consequently, it is now expected that Indigenous research will be community based and community participatory,^18^ meaning that it will reflect the needs of the community and offer something in return, which may be anything from health services to local workforce development.

A more participatory model requires researchers to interact with Indigenous communities over a period of time. It is the researcher’s responsibility to learn about the community’s social and political structure,^19^ although there is some responsibility on the community’s part to assist the researchers in gaining understanding of culture and their requirements. This may take time, and an initial social invitation to participate in community activities may develop into a more formal relationship.^12^

Importantly, once this relationship is formed it must be viewed as long term and possibly permanent. A common community criticism of researchers is that results are never reported back^19^ and that once funding or the project ceases, the community is left in limbo. Successful research groups have used regular scheduled visits^11^ and newsletters^10,16^ to maintain community contact and engagement. Recruitment of Indigenous community members to assist with engagement has been shown to increase research recruitment and act as an important conduit to maintain direct community input^22^; however, researchers must be conscious of not placing community members in positions of cultural and social conflict with their requests.^22^

Finally, community participation in research often incurs financial costs for the community. These must be recognized and reimbursed.^15^ Although Western research models are wary of offering financial and other inducements for participation,^19^ the Human Genome Organization stated that “undue inducement through compensation for individual participants, families and populations should be prohibited. This prohibition does not include agreements with individuals, families, groups, communities or populations that foresee technology transfer, local training, joint ventures, provision of health care or of information, infrastructures, reimbursement of costs, or the possible use of a percentage of any royalties for humanitarian purposes.”^25^

## DISCUSSION

The development of tissue banks and biorepositories is now part of the US National Cancer Institute standards of best practice,^2^ and most medical institutions have at least one. There is now also increasing cooperation between biobanks, particularly related to sharing of tissues, to assist researchers in the study of rare conditions, although this is not legislatively mandated. Consequently, examples of best practice governance models for biobanks are sought after as part of ongoing quality improvement to increase donor recruitment and avoid negative experiences. Tissues and specimens from Indigenous peoples continue to be targeted to increase understanding of risk and protective factors for many conditions.

This review can be criticized for combining data on multiple Indigenous peoples (New Zealand Māori, Aboriginal, Torres Strait Islanders, Polynesian, and First Nations), attempting to homogenize peoples and possibly oversimplifying socially and geographically unique cultures. However, when information from each culture was reviewed, it was remarkable how similar many of the founding beliefs were, how similar the experiences of many Indigenous peoples with Western researchers were, and how many Indigenous peoples shared the same concerns with tissue and biobanking practices. Consequently, a thematic approach was taken to select four themes that were present in literature from all Indigenous communities. In addition, only a relatively small number of reports exist, and only in five specific population groups, describing Indigenous peoples’ experience with biobanking. All reported investigations are qualitative and are based on the experiences of small numbers of participants. Consequently, although the investigations reported in this review had a low or unclear level of bias as assessed using the EPOC tool, bias may have been present in participant recruitment, with those most dissatisfied with their experience more likely to participate in assessment and review

For Western researchers, the hardest theme to comprehend may be the connection of tissue to ancestral lands, ancestors, and culture, with the understanding that in asking for tissue donation they may be asking for much more. Such a request must therefore be based on a solid and trusting community relationship, and this will need to be developed and nurtured over time. Once established, the community may well view it as permanent and expect it to be enduring. However, in Western society there are similar findings, where potential tissue donors prefer to have the process explained and to be asked by a person well-known and trusted by them (often, but not always, a physician).^2,26^ Similarly, all cultures—both Western and in the developing world—want research to take account of their religious and cultural beliefs and for research to not contradict these values.^27,28^ Consequently, a solid community relationship must underpin any biobanking research.^29^ Indigenous communities are increasingly well connected and qualified and wish to take part in advanced medical research as a means of improving their own health and contributing to the society in general.^3,9^ Indigenous communities can bring important research questions and other resources, such as detailed family pedigree information, to any genomic project.

Studies of biobanking donor concerns in Western society highlight issues of consent and specimen ownership, as do those from Indigenous communities. In multiethnic investigations, most potential donors were comfortable with the concept of biobanking if informed consent and confidentiality could be assured.^2^ However, up to two-thirds of donors would prefer to be reconsented for any new use of donated tissue,^2,30^ and many donors have concerns about the loss of control of personal data that could accompany biobanking in an era where retaining personal control over personal data is emphasized.^31^ However, many Western donors, regardless of ethnicity, are willing to forego confidentiality of biospecimen collection to receive potential therapeutic benefit to themselves or to family members.^30,32^ Not surprisingly, the opinions of many Western donors on the optimal form of consent are split, with specific, tiered, or open consents preferred by different people in a review of optimal consent practice from the United Kingdom.^33^ However, this may vary with the community studied; a detailed investigation of a multiethnic community in the United States found that no donors, regardless of ethnicity or age, wanted to reconsent specimen use when new research projects were suggested.^34^ In turn, this finding may represent a consequence of acculturation. In a study of attitudes to biobanking in a Korean American community, members were more likely to donate if they had lived in the United States for a long time, had private health insurance, and had an annual income of greater than US$40,000.^35^ Similarly, in the United Kingdom, donors with a tertiary education level and some knowledge of medical science were more likely to donate without reservation,^36^ suggesting that familiarity with Western medical science and previous positive experience of Western medicine are strong promotors of tissue donation.

## RECOMMENDATIONS

### Theme 1: Land, Ancestors, Culture, and Bodily Substances Are Powerfully Interconnected and Can Act on Each Other

(1) The primary responsibility of the researchers is to gain an understanding of the social and political aspects of Indigenous communities and an appreciation of their belief systems to understand where the proposed research can fit within the community. Ideally, the community should work with the researcher and team to optimize this. (2) The research must be relevant and contribute to the well-being and/or the development of the community. (3) There is need for an internationally agreed legislative framework to cover the rights of Indigenous peoples engaging in biobanking and genomic research. (4) Careful consideration must be applied to any requirements to remove biologic specimens from community lands or the country of origin for the purposes of analysis.

### Theme 2: Tissue and Blood Can Provide Important Information (Both Western and Traditional) About a Person

(1) All Indigenous biospecimens are precious and must be handled in a way that does not contradict community beliefs and standards. Community-led expert advisory groups can assist the research team with this process. (2) Research results should be shared with the community before any widespread dissemination. (3) Any form of genetic modification is fraught and must be formally and openly discussed.

### Theme 3: The Ownership of Specimens—Custodians, Trustees, or Guardians?

(1) Specimens and knowledge gained from the project ideally should remain under the ownership of the community but under the stewardship of the research team. (2) The use of anonymized specimens in Indigenous research requires special consideration and, paradoxically, may not be wanted by communities. Consideration should be given to returning research results to individuals and their families. (3) Retaining or returning biospecimens after death of an Indigenous donor must be specifically discussed as part of the consenting process.

### Theme 4: The Beneficence of the Researchers and Research Team

(1) The research team must prioritize the welfare of the Indigenous community at all times. (2) Good research will require the development of a long-lasting and trusting relationship between the community and the research team. (3) Regular communication must be maintained between the research team and the community by face-to-face meetings, newsletters, or other forms of communication. (4) Costs to the communities for research participation must be recognized and reimbursed.

## CONCLUSION

Review of the barriers to participation by Indigenous communities in biobanking and genomic research has demonstrated that Indigenous communities, like Western populations, are concerned with issues around the handling, treatment, and ownership of tissue and knowledge gained from specimen analysis. Unlike many Western populations, Indigenous communities have retained a strong sense of cultural connection to ancestors and traditional lands and view biologic specimens as inseparable from these things. The challenge for researchers is to demonstrate that improvements in community culture and well-being can be achieved with knowledge gained from biobanking and genomic research.
